# Supplementary material for: Investigating the Effects of Donors and Alkyne Spacer on the Properties of Donor-Acceptor-Donor Xanthene-Based Dyes
Source: Molecules. 2023 Jun 22;28(13):4929. doi: 10.3390/molecules28134929 (PMC10343496; doi:10.3390/molecules28134929)
Supplement: Supplementary file 1 [file molecules-28-04929-s001.zip › molecules-2406525-supplementary.pdf]

Investigation of donors and alkyne spacer on the properties of donor-acceptor-donor xanthene-based dyes

Ishanka N. Rajapaksha[1], Jing Wang,[2] Jerzy Leszczynski,[2] and Colleen N. Scott[1*]

Supporting information

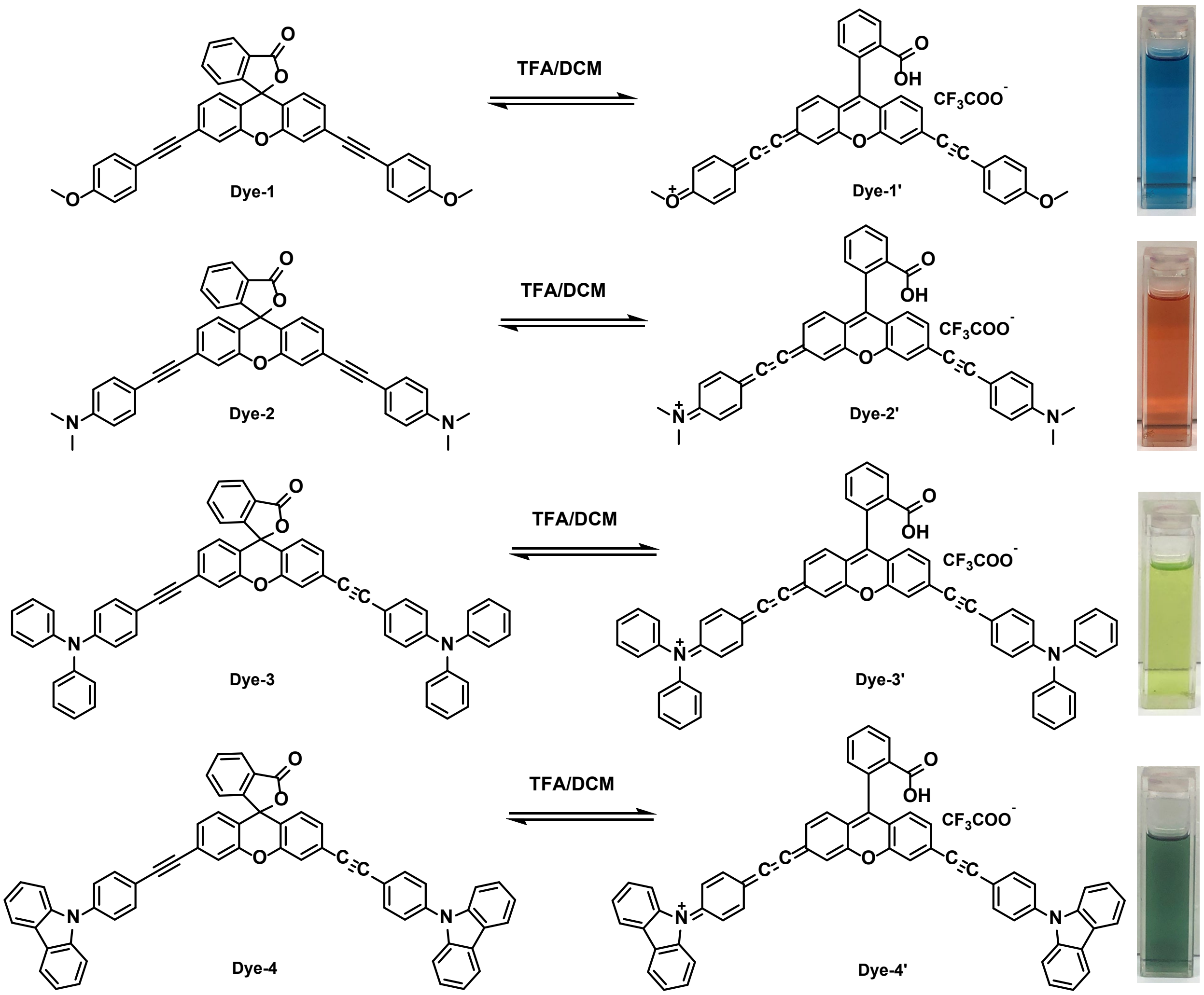

Figure S1 Structures and colors of **Dyes 1-4** after opening with TFA.

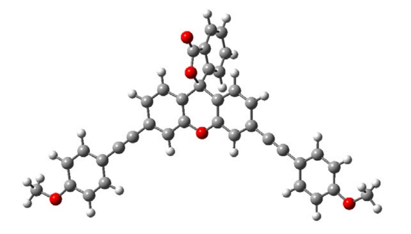

Dye 1 Closed

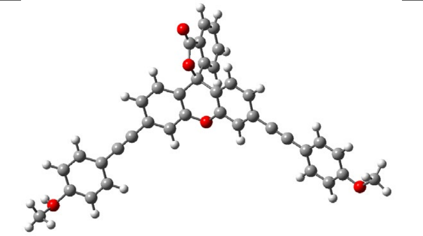

Dye 1 H$^+$ Closed

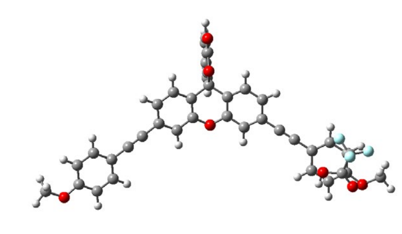

Dye 1 Closed + CF_3COOH

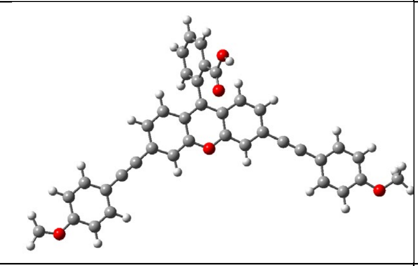

Dye 1 Opened

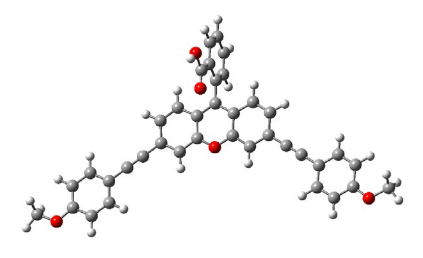

Dye 1 H$^+$ Opened

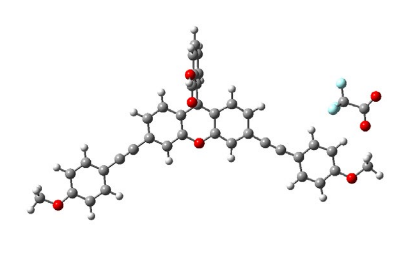

Dye 1 Opened + CF_3COOH

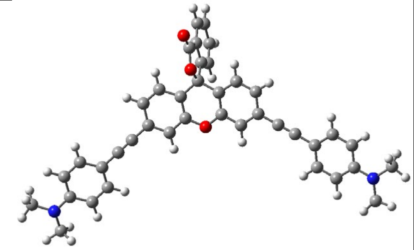

Dye 2 Closed

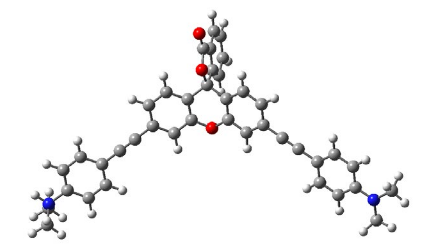

Dye 2 H$^+$ Closed

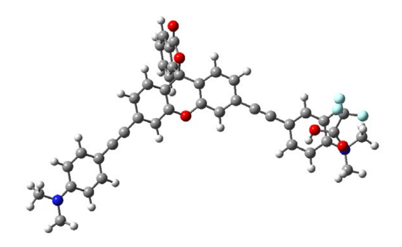

Dye 2 Closed + CF_3COOH

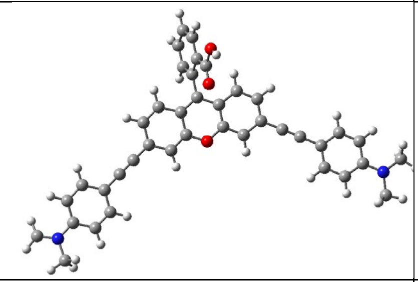

Dye 2 Opened

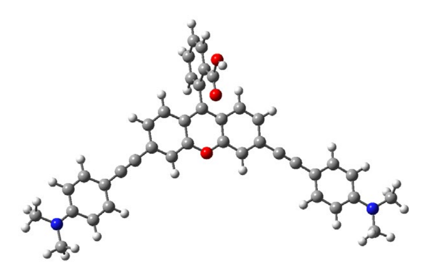

Dye 2 H$^+$ Opened

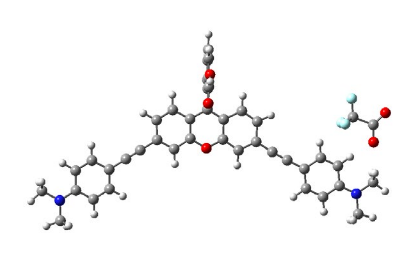

Dye 2 Opened + CF_3COOH

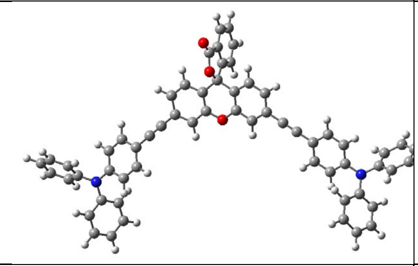

Dye 3 Closed

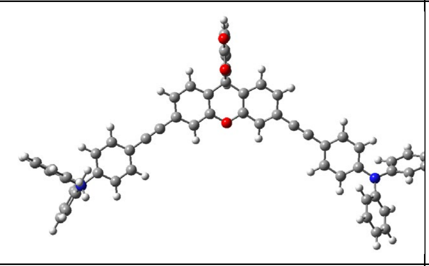

Dye 3 H$^+$ Closed

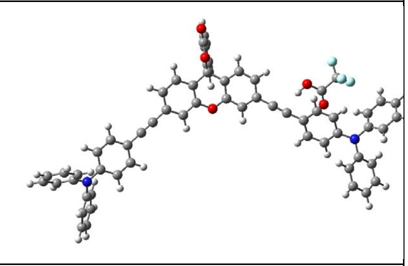

Dye 3 Closed + CF_3COOH

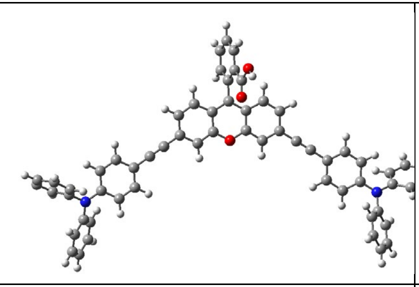	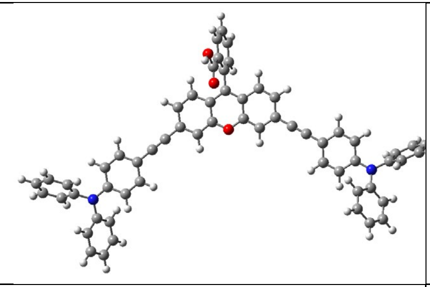	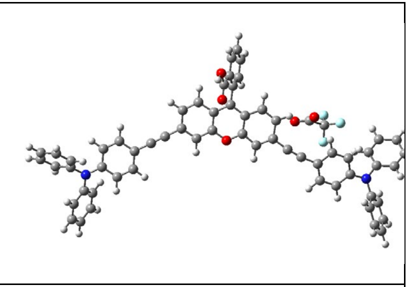
Dye 3 Opened	Dye 3 H^+ Opened	Dye 3 Opened + CF_3COOH
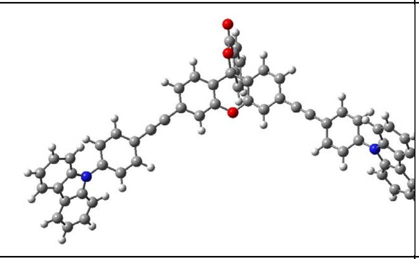	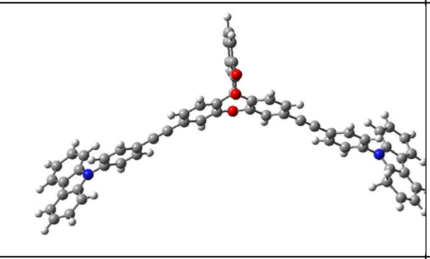	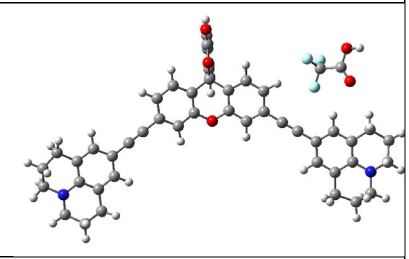
Dye 4 Closed	Dye 4 H^+ Closed	Dye 4 Closed + CF_3COOH
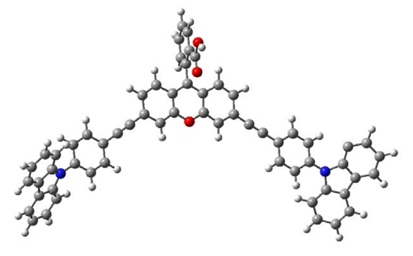	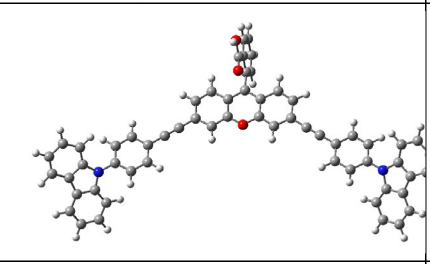	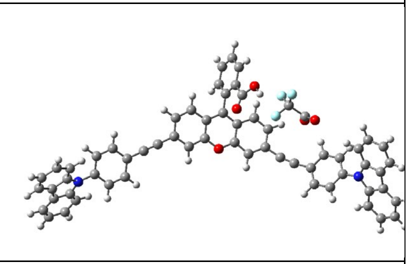
Dye 4 Opened	Dye 4 H^+ Opened	Dye 4 Opened + CF_3COOH

Figure S2. Optimized structures in gas phase with neutral form, positive charged form, and CF_3COOH combined complex (M06-2X/6-311G(d,p) level)

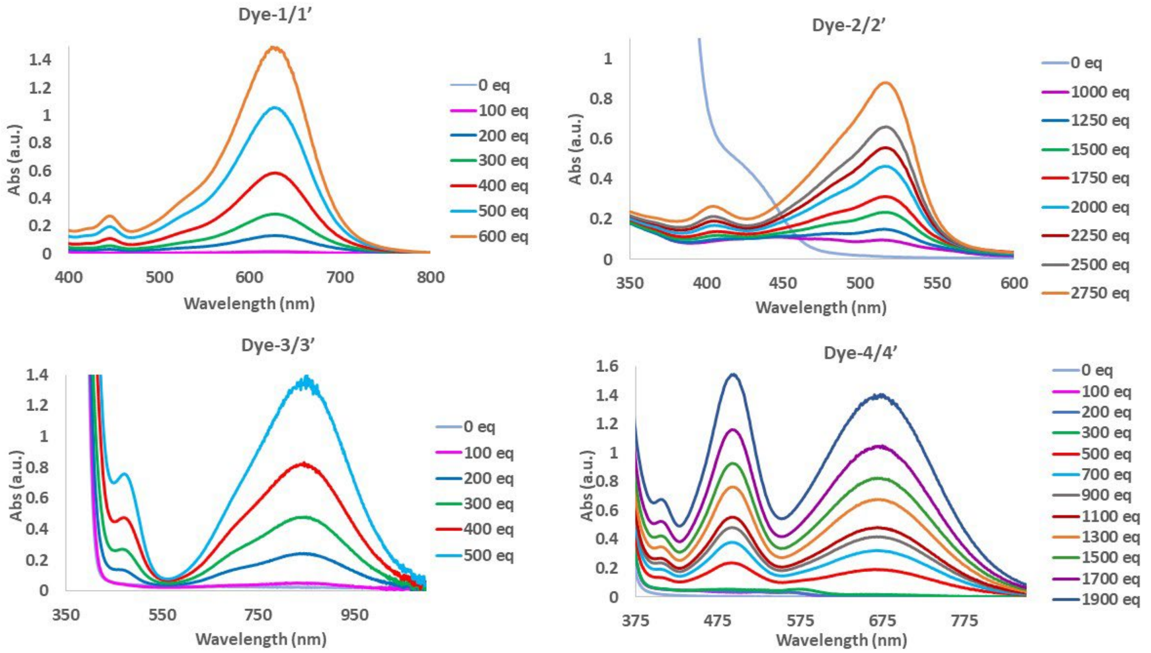

Figure S3. Opening of **Dyes 1-4** with different equivalents of TFA

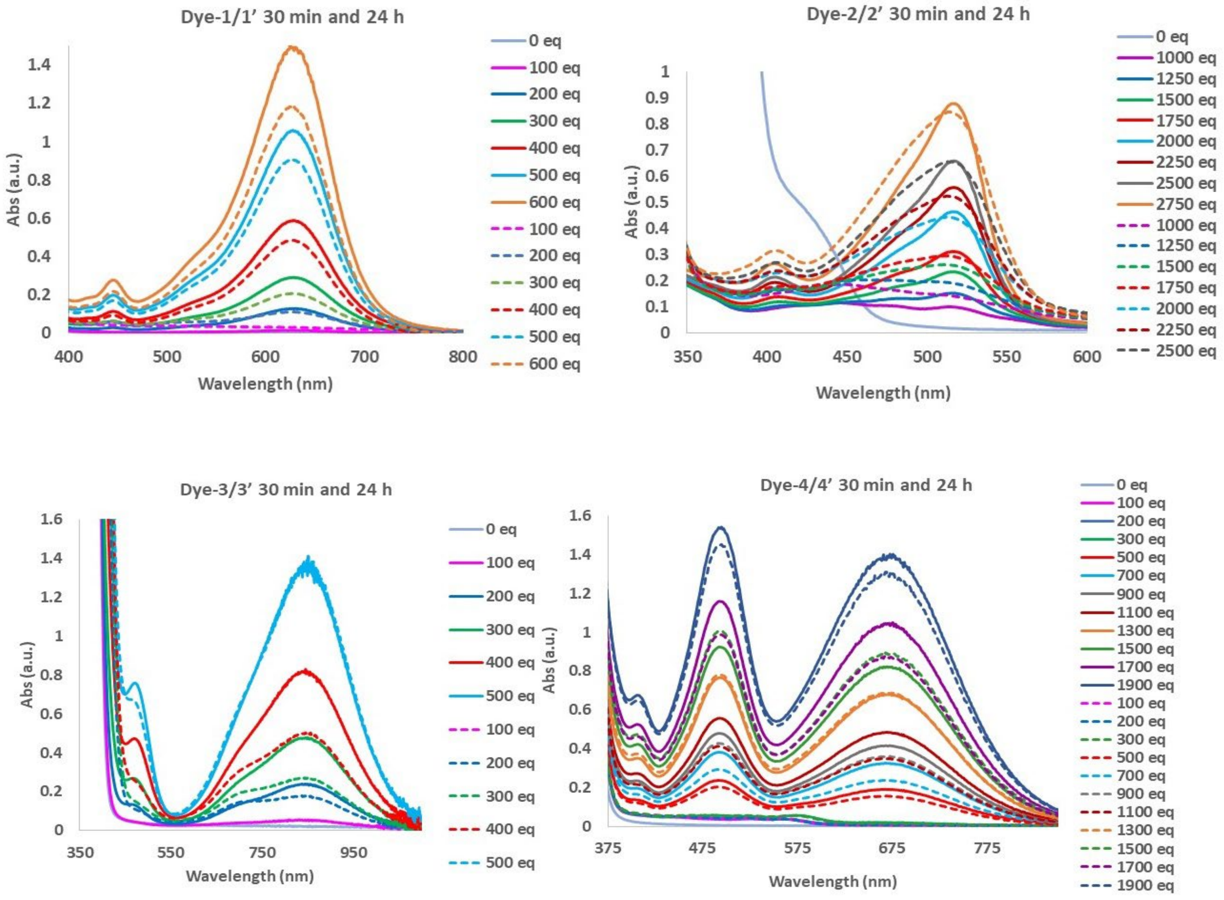

Figure S4. Equilibration of the dyes for 24 h with different equivalents of TFA. 30 mins (solid lines); 24 h (dashed lines).

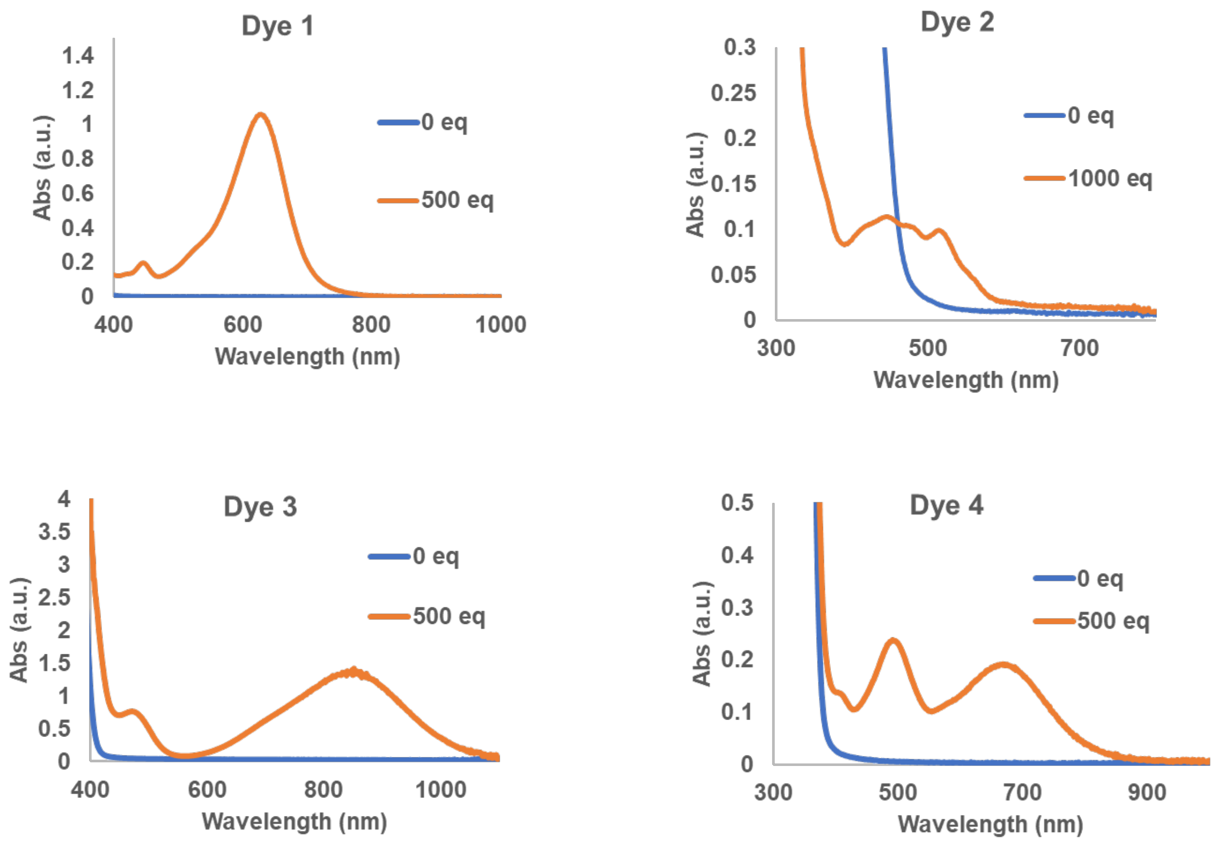

Figure S5. Apsorption spectra of **Dyes 1-4** in closed (0 eq TFA) and opened (500 or 1000 eq TFA) that was used to calculate the difference on the absorbance intensity between the closed and opened forms.

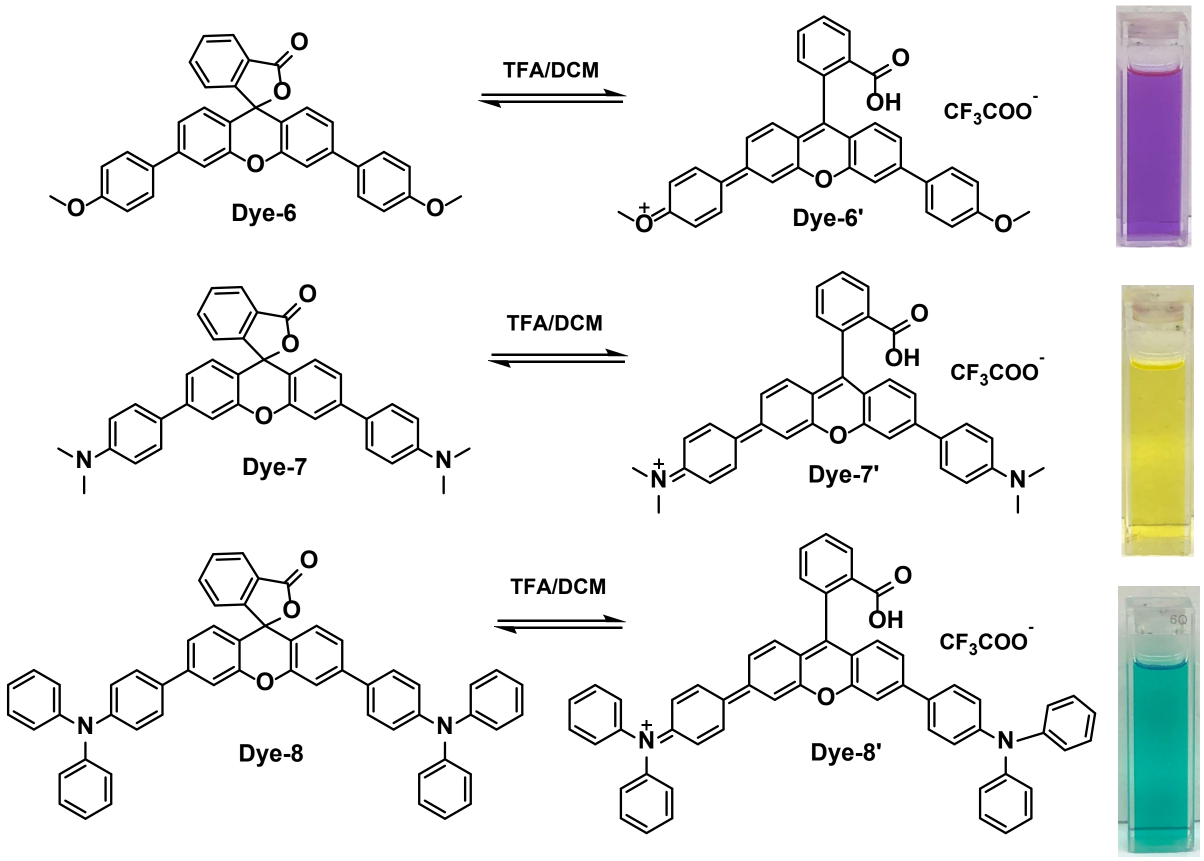

Figure S6. Structures and colors of **Dyes 6-8** after opening with TFA.

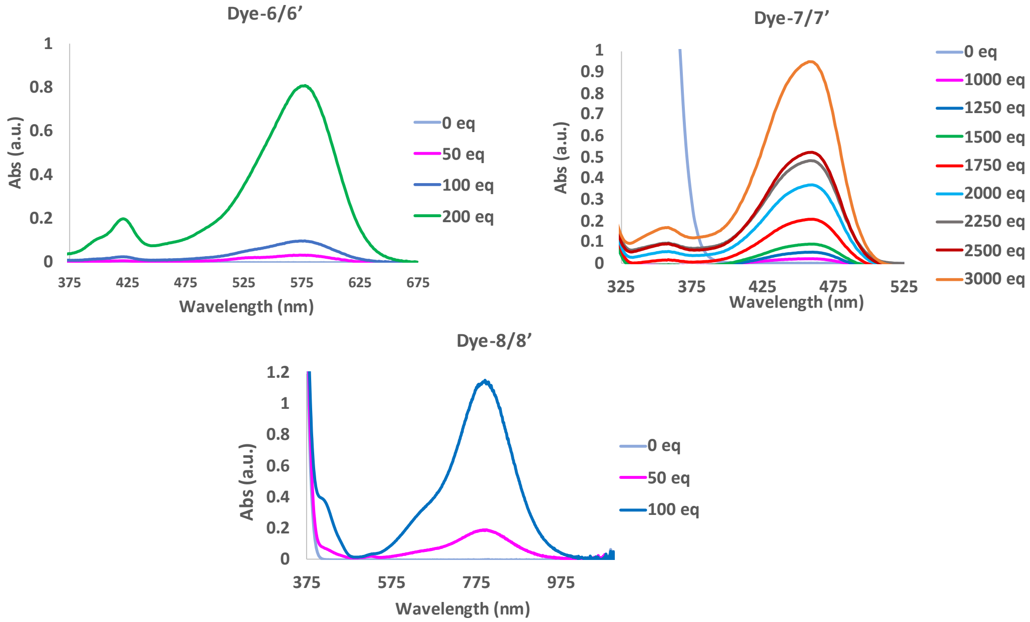

Figure S7. Opening of **Dyes 6-8** with different equivalents of TFA.

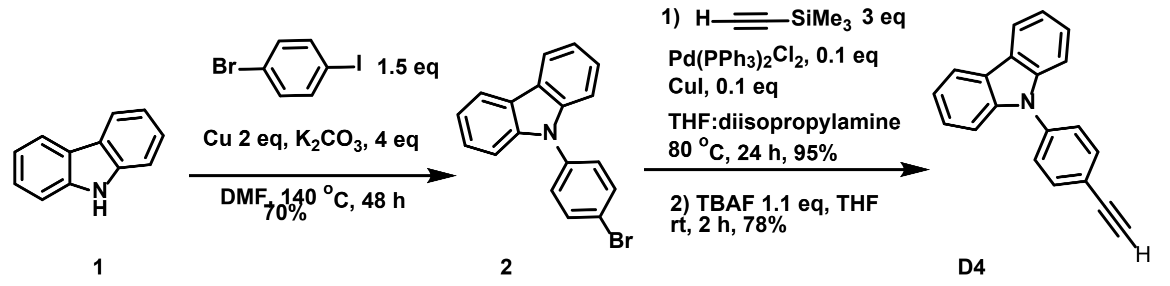

Scheme S1 Synthesis of **D-4** compound

Synthesis of 9-(4-ethynylphenyl)-9H-carbazole (D4)

9-(4-bromophenyl)-9*H*-carbazole[51] (Compound 2) (0.3104 mmol, 100 mg), $PdCl_2(PPh_3)_2$ (0.0310 mmol, 21.8 mg), and CuI (0.0310 mmol, 5.90 mg) were added into a dried microwave vial in the glovebox. THF (0.5 mL), diisopropylamine (1.5 mL), and TMS-acetylene (1.552 mmol, 0.23 mL) were added to the vial under nitrogen atmosphere and the reaction mixture was stirred at 80 °C for 24 h. The reaction mixture was allowed to come to room temperature and diluted with DI water (30 mL) and DCM (20 mL). The layers were separated, and the aqueous phase was extracted with DCM (3 x 20 mL). The combined organic layer was washed with 0.1 M HCl, saturated sodium bicarbonate solution, dried over anhydrous sodium sulfate, filtered, and concentrated under reduced vacuum. The crude was purified by flash column chromatography using hexanes as the eluent to give 9-(4-((trimethylsilyl)ethynyl)phenyl)-9H-carbazole product as a white solid in 95 % yield. **^{1}H NMR** (500 MHz, $CDCl_3$) δ 8.17 (d, *J* = 5.6 Hz, 2H), 7.73 (d, *J* = 8.5 Hz, 2H), 7.55 (d, *J* = 8.4 Hz, 2H), 7.47 – 7.41 (m, 4H), 7.33 (m, *J* = 5.2, 2.5 Hz, 2H), 0.34 (s, 9H).

9-(4-((trimethylsilyl)ethynyl)phenyl)-9H-carbazole (0.2945 mmol, 100 mg) and THF (2 mL) were added into a dried round bottom flask. To the reaction mixture was added tetra-n-butylammonium fluoride (TBAF) (1M in THF, 0.3534 mmol) under nitrogen atmosphere for 2 h. The reaction mixture was diluted with DI water 30 mL and DCM 20 mL. The layers were separated, and the aqueous phase was extracted with DCM (3 x 20 mL). The combined organic layer was washed with 0.1 M HCl and saturated sodium bicarbonate solution, dried over anhydrous sodium sulfate, filtered, and concentrated under reduced pressure. The crude was purified by flash column chromatography using hexanes as the eluent **D4** as a white solid in 78 % yield. **^{1}H NMR** (500 MHz, $CDCl_3$) δ 8.14 (d, *J* = 7.8 Hz, 2H), 7.73 (d, *J* = 8.5 Hz, 2H), 7.56 (d, *J* = 8.5 Hz, 2H), 7.42 (d, *J* = 2.4 Hz, 4H), 7.34 – 7.29 (m, 2H), 3.18 (s, 1H).

^{1}H NMR of compound Dye-1 (500 MHz)

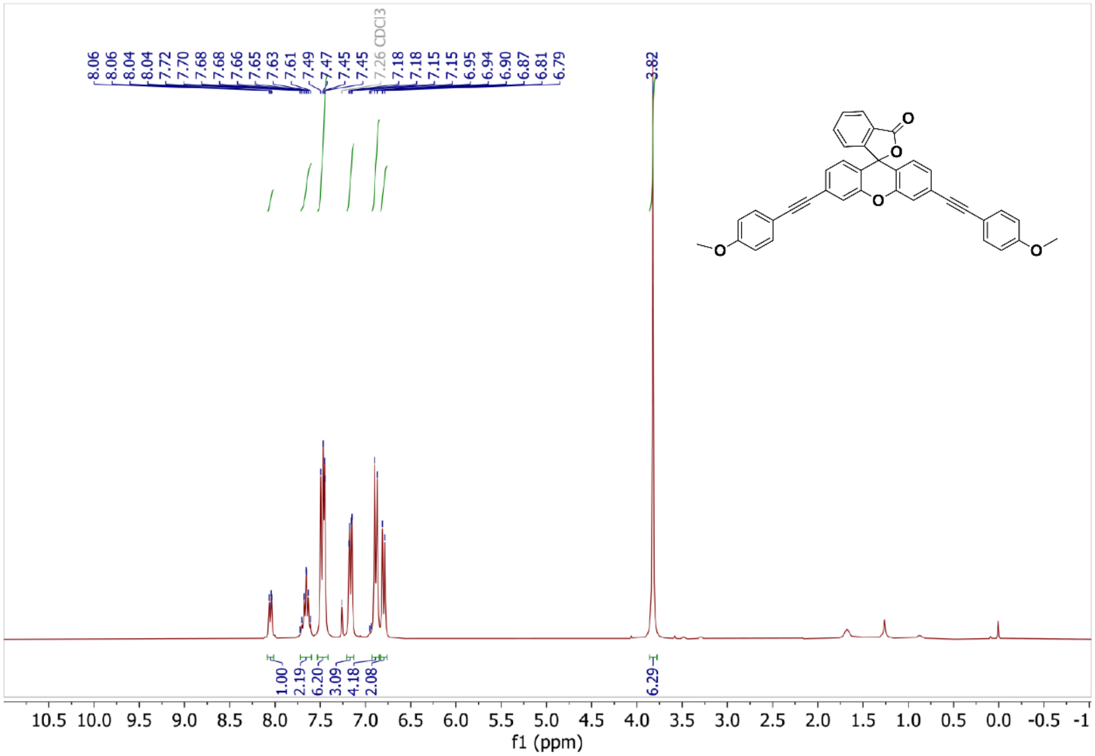

^{13}C NMR of compound Dye-1 (125 MHz)

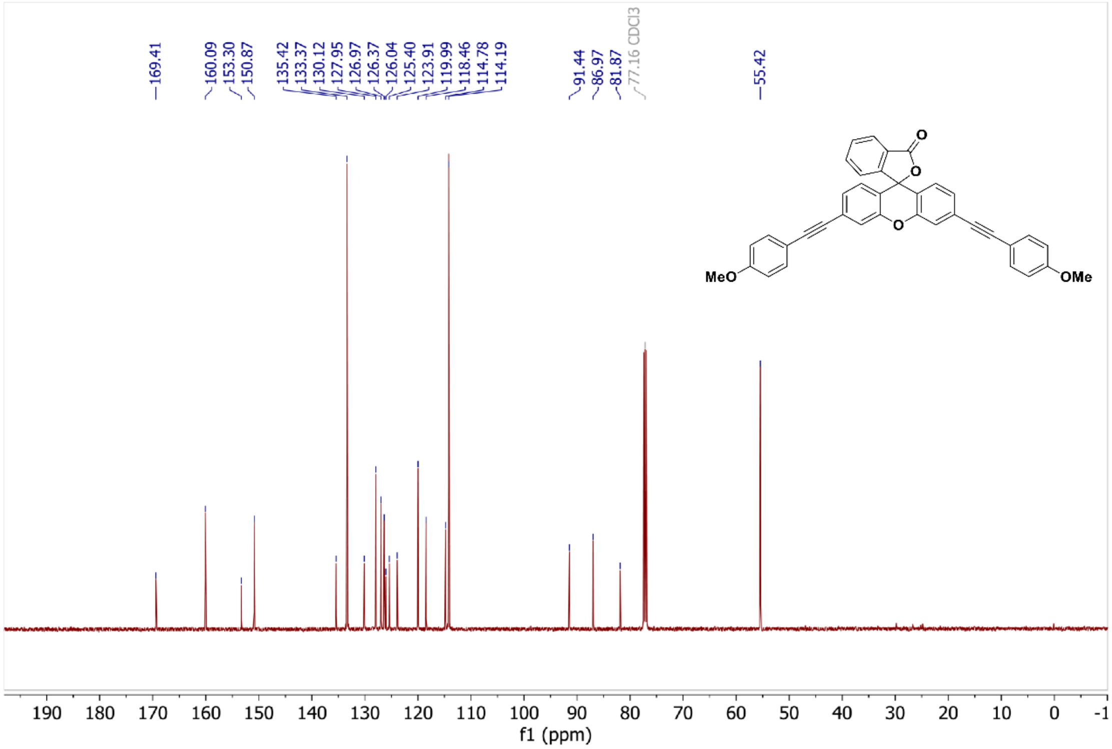

^{1}H NMR of compound Dye-2 (500 MHz)

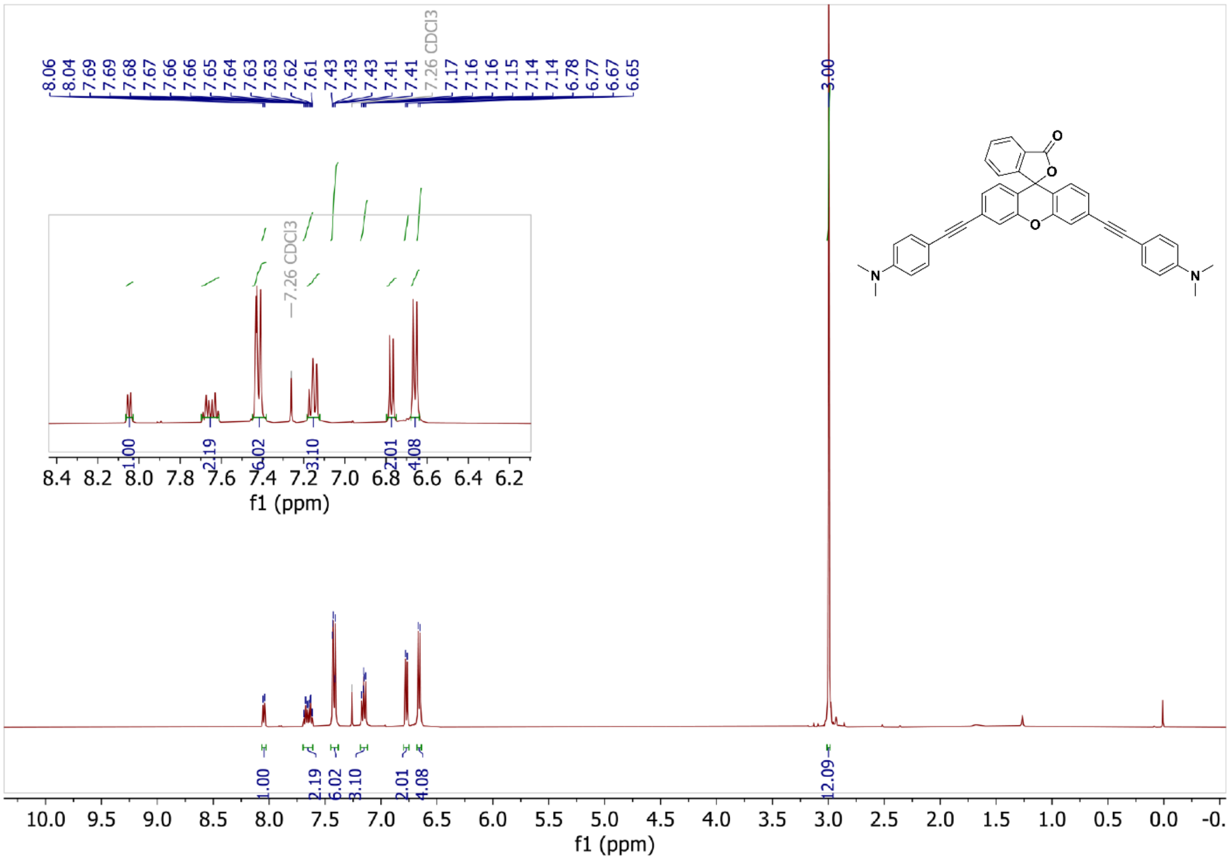

^{13}C NMR of compound Dye-2 (125 MHz)

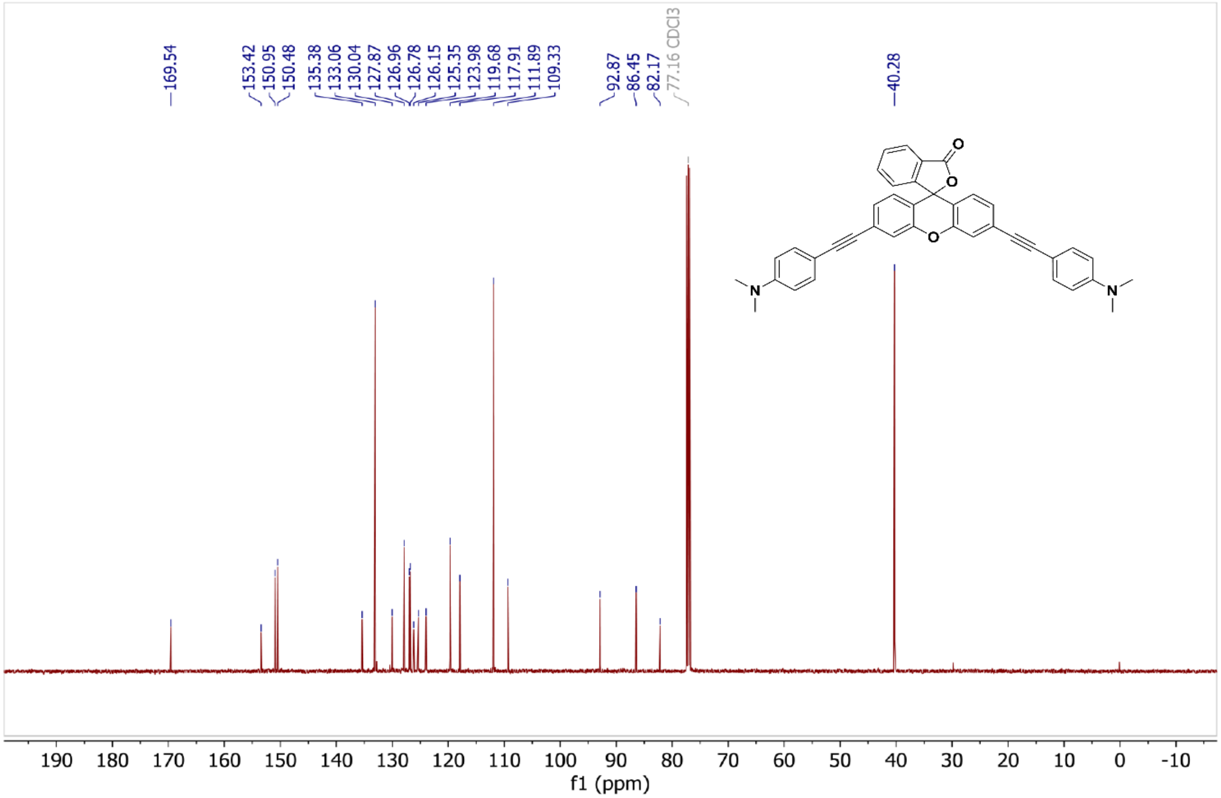

^{1}H NMR of compound Dye-3 (500 MHz)

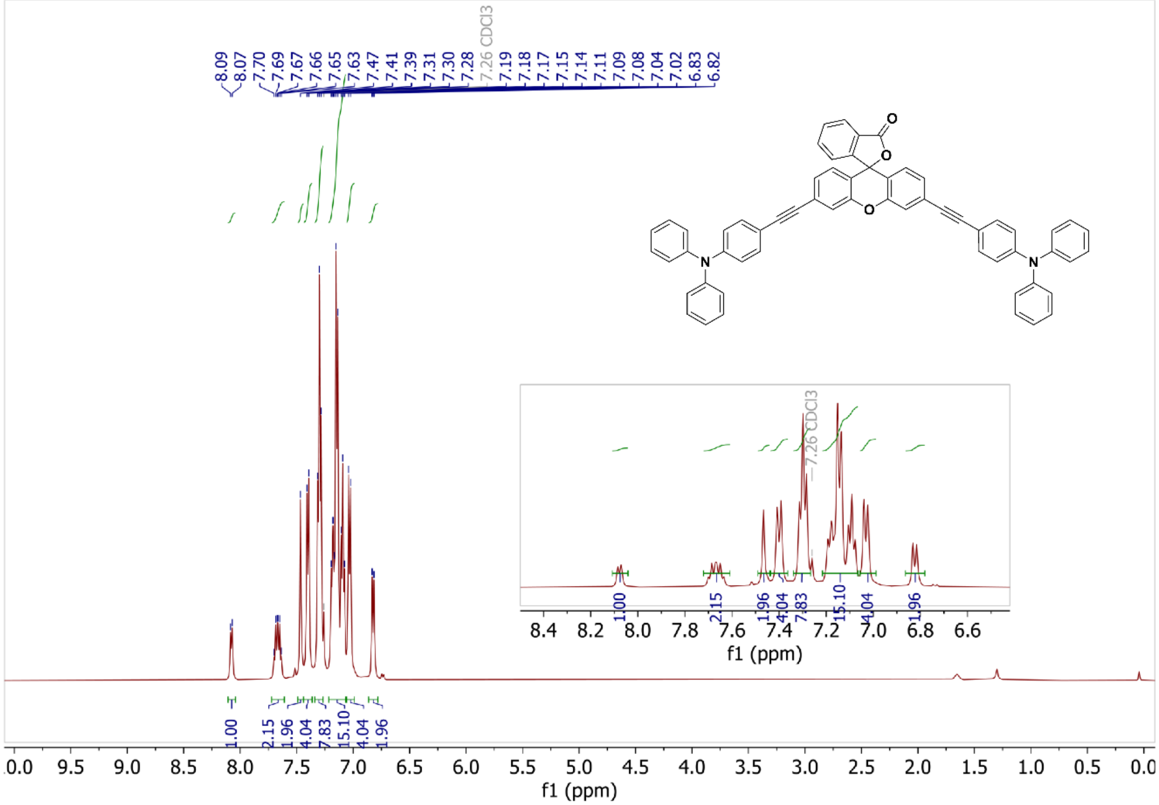

^{13}C NMR of compound Dye-3 (125 MHz)

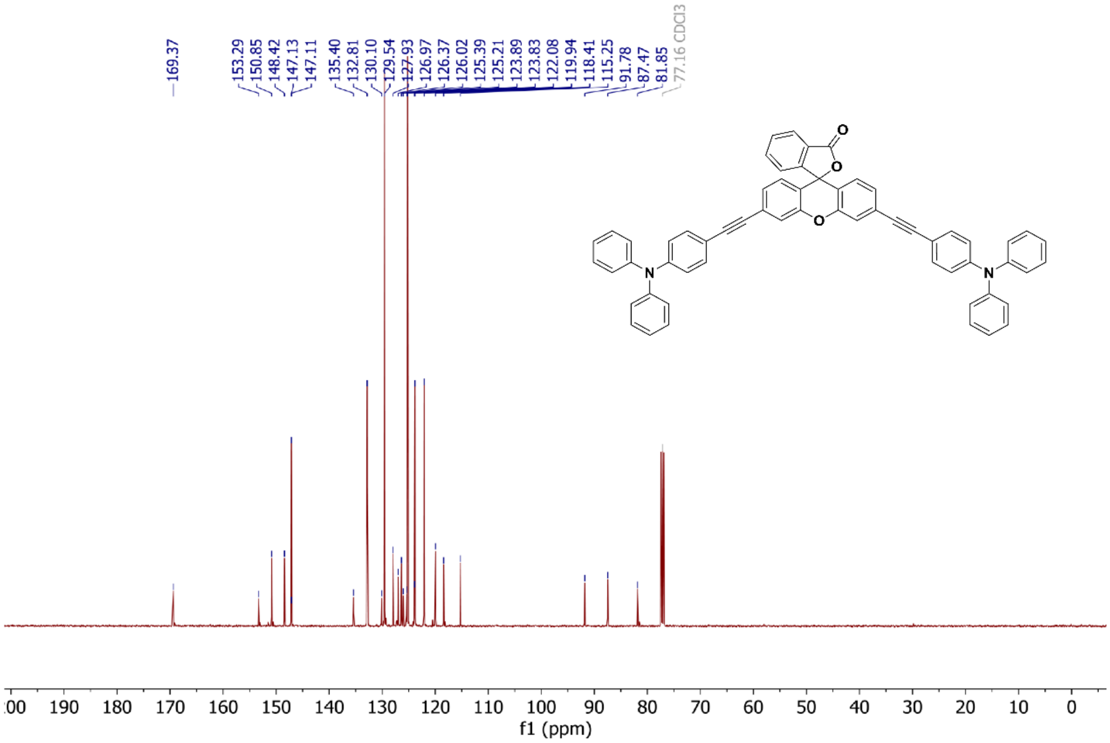

^{1}H NMR of compound Dye-4 (500 MHz)

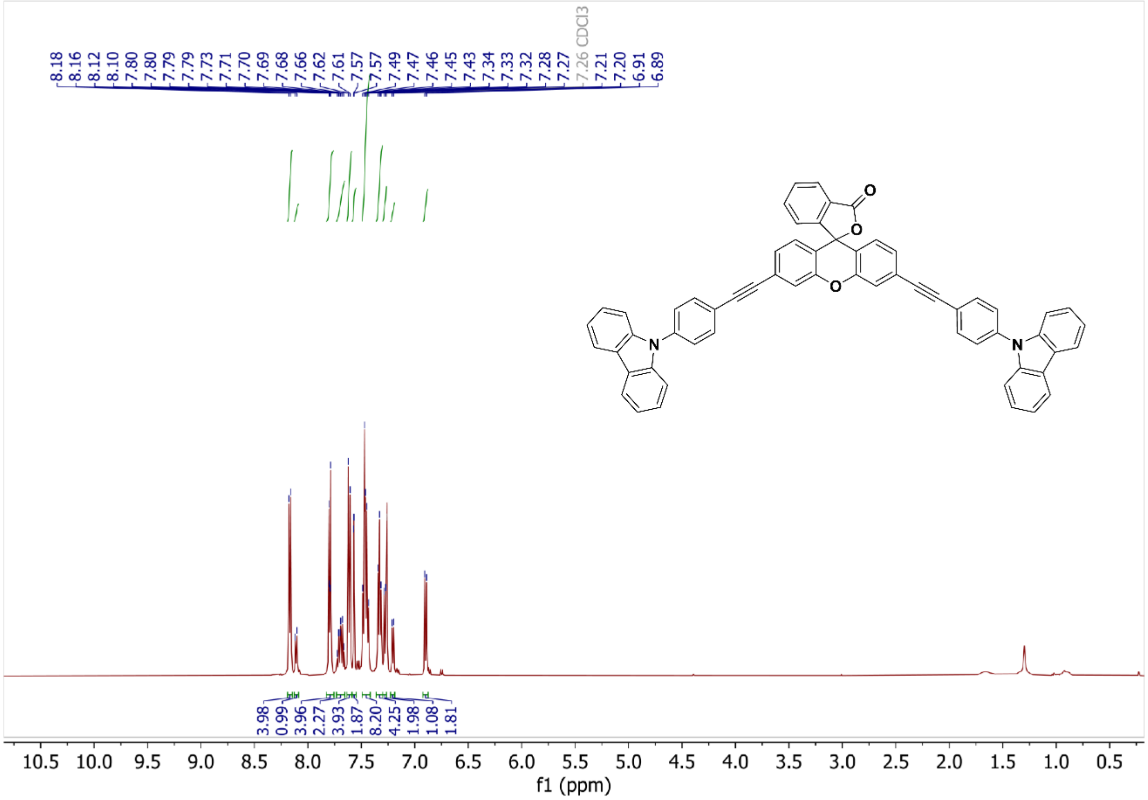

^{13}C NMR of compound Dye-4 (125 MHz)

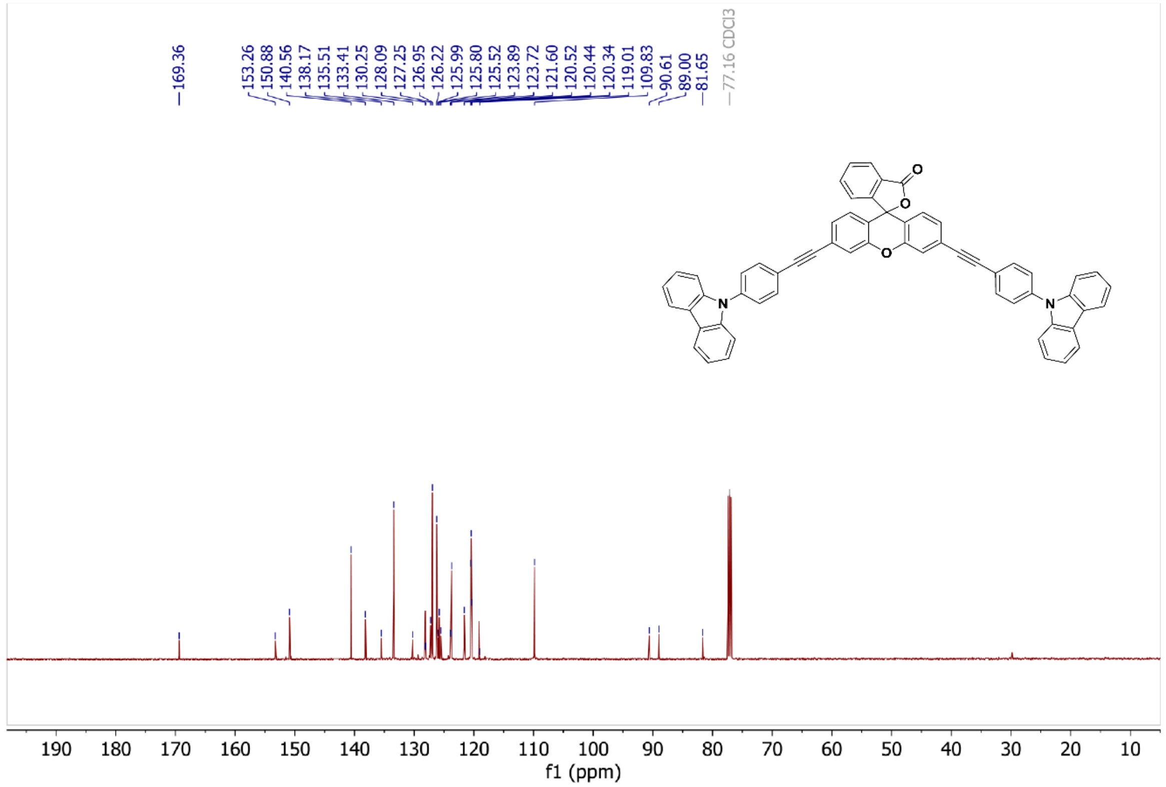

^{1}H NMR of compound Dye-6 (500 MHz)

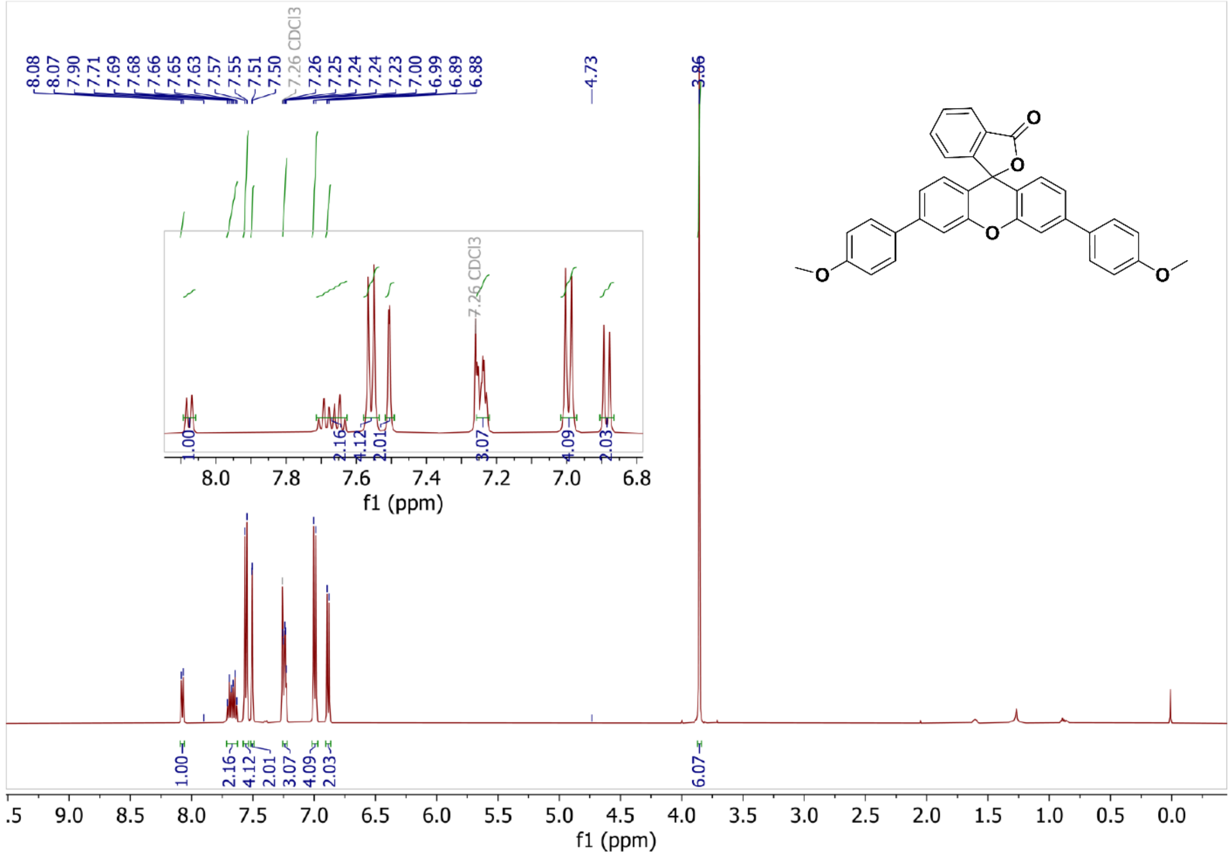

^{13}C NMR of compound Dye-6 (125 MHz)

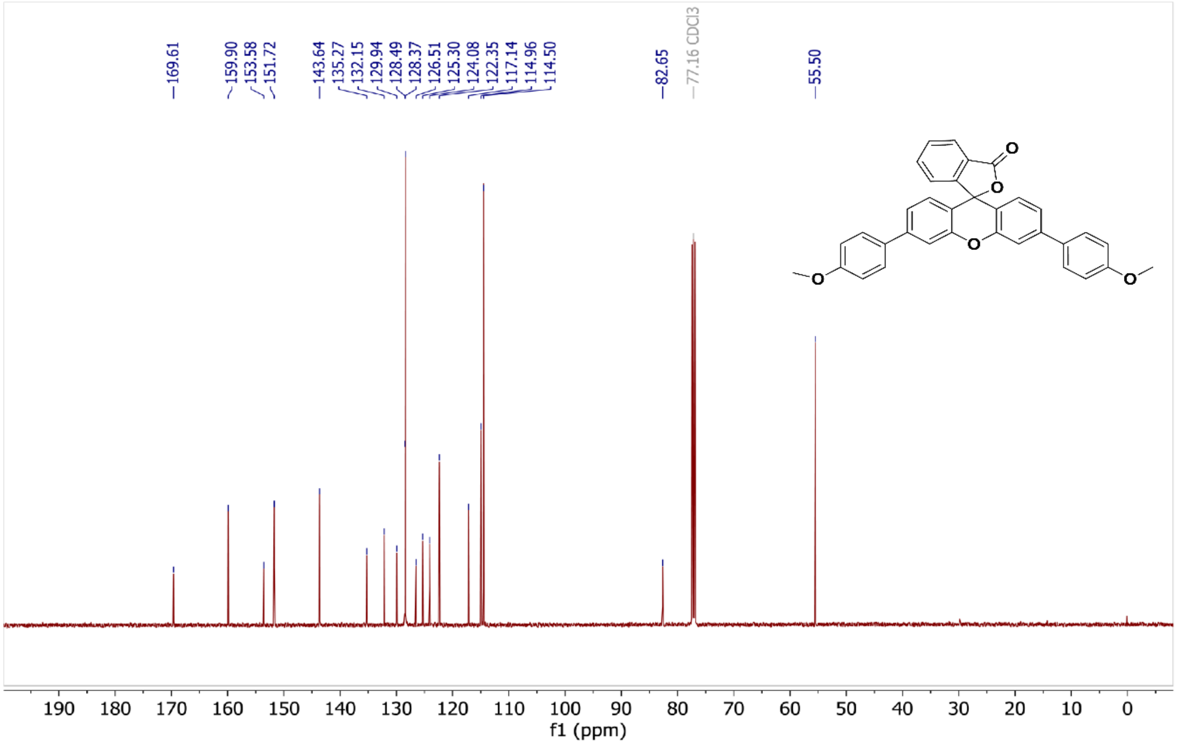

^{1}H NMR of compound Dye-7 (500 MHz)

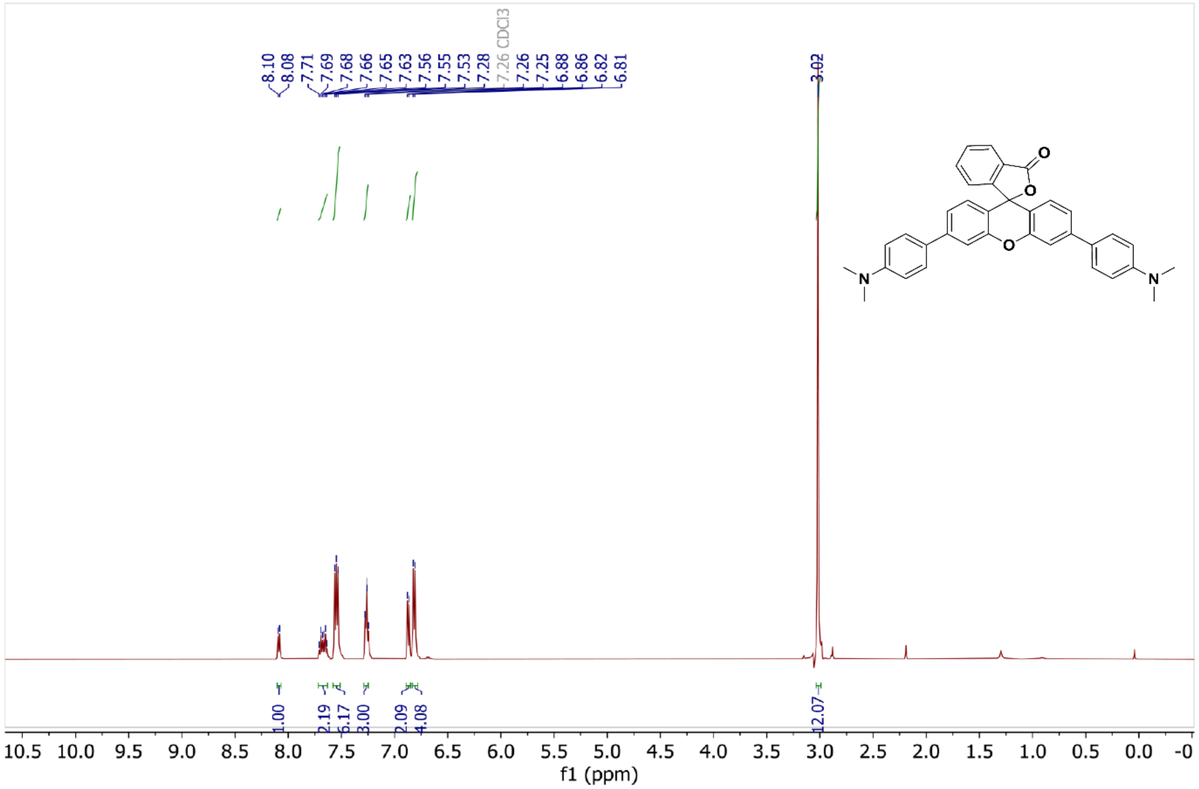

^{13}C NMR of compound Dye-7 (125 MHz)

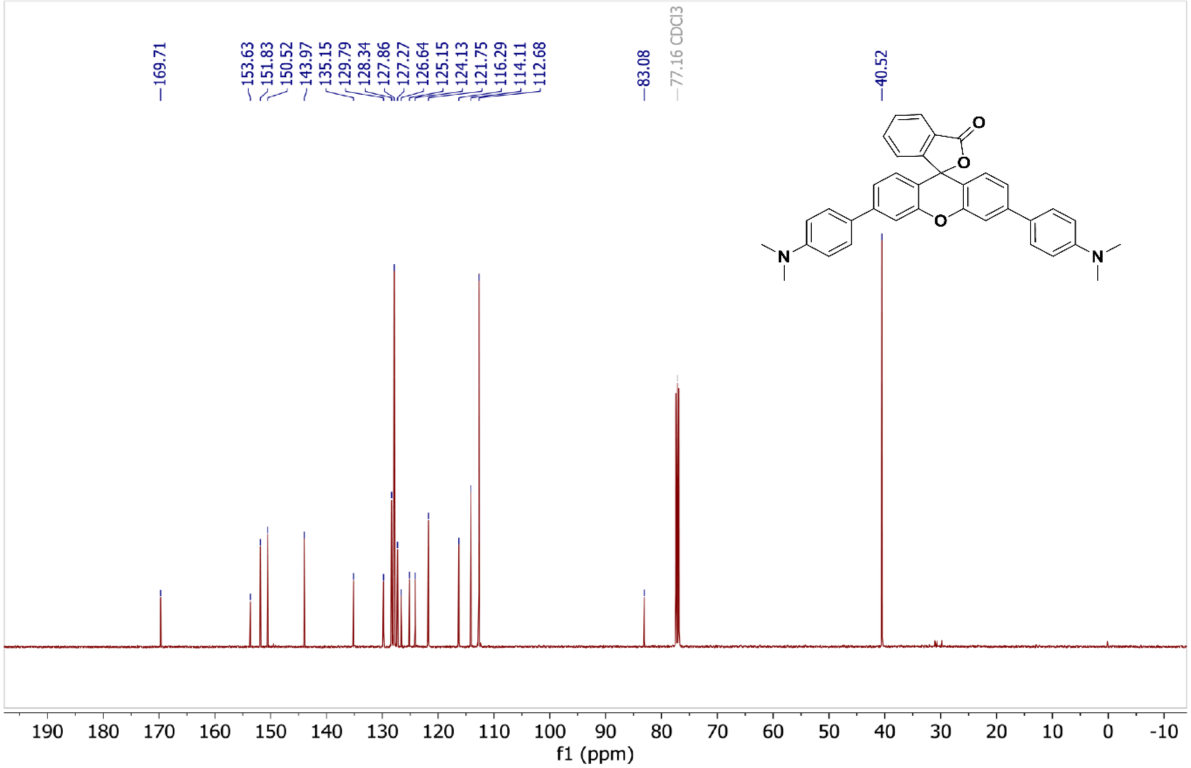

^{1}H NMR of compound Dye-8 (500 MHz)

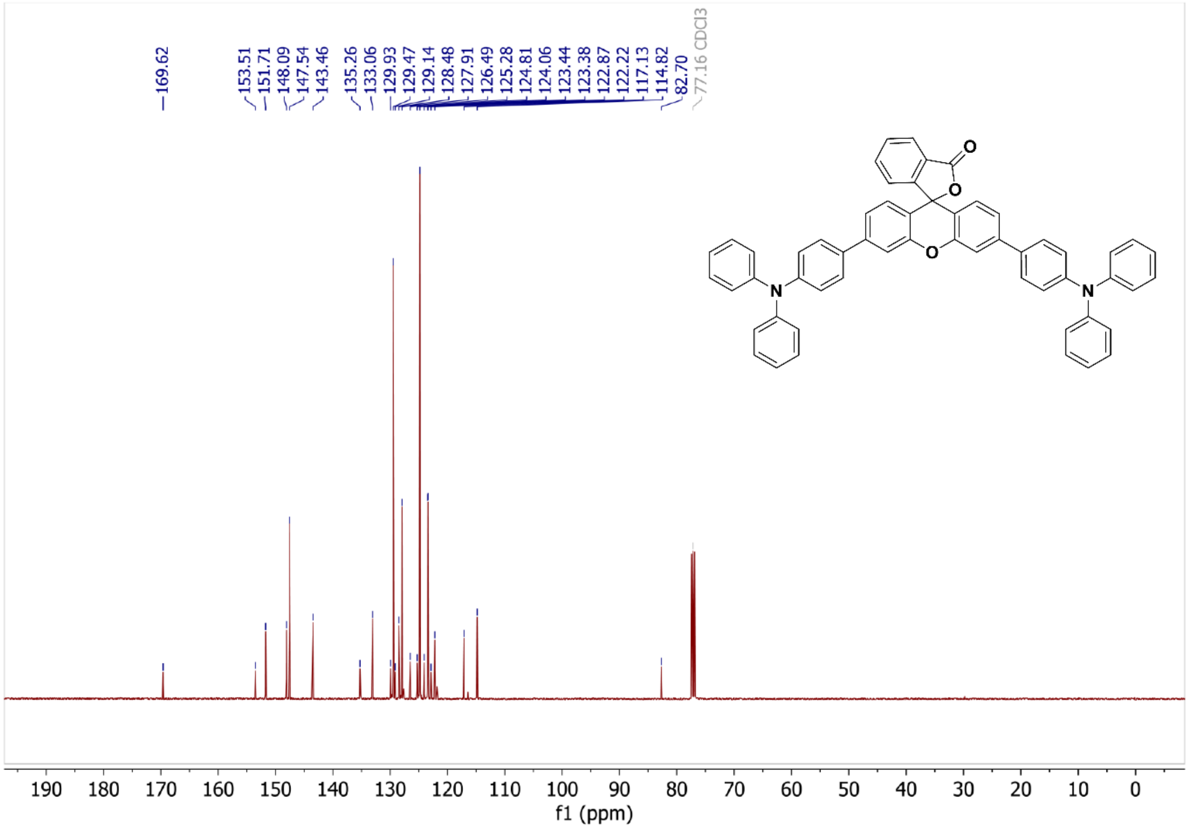

^{13}C NMR of compound Dye-8 (125 MHz)

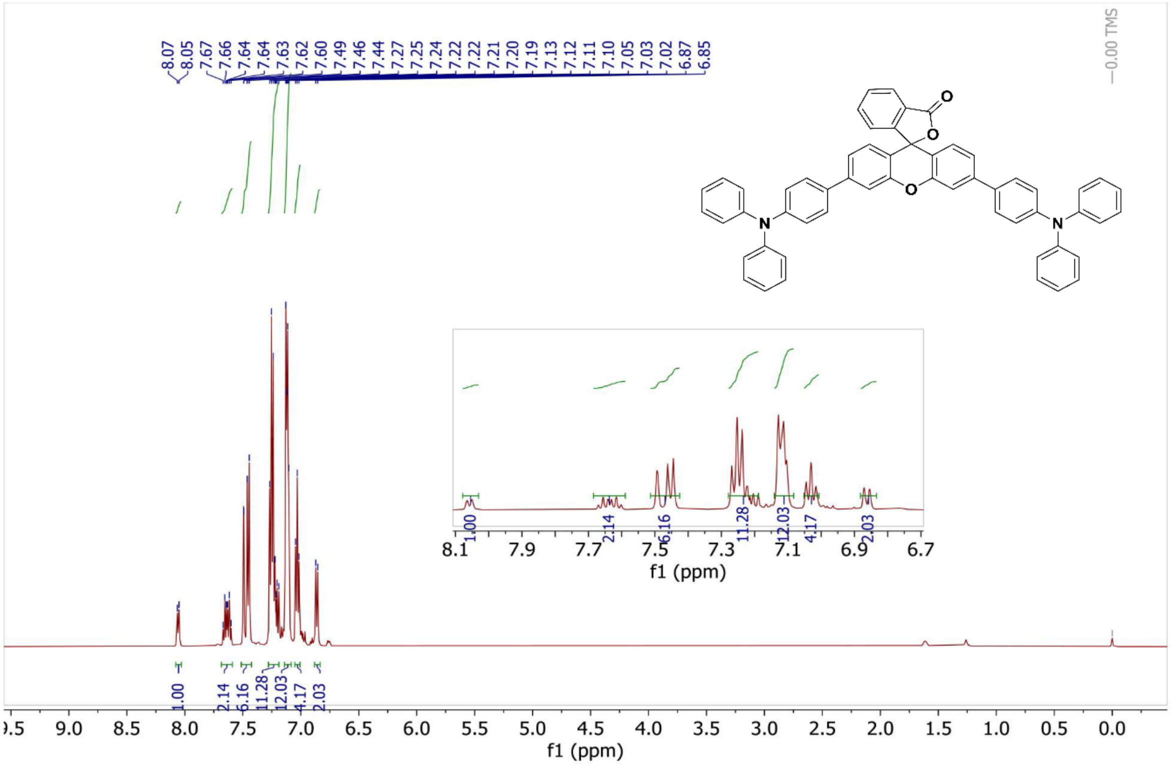

HRMS Analysis (Dyes 1-4 and 6-8)

Dye-1

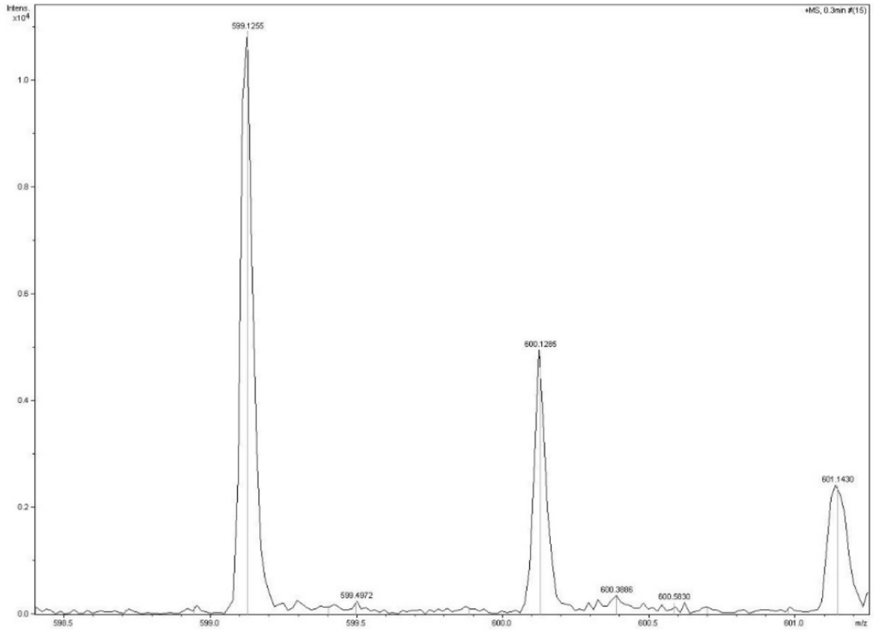

Dye-2

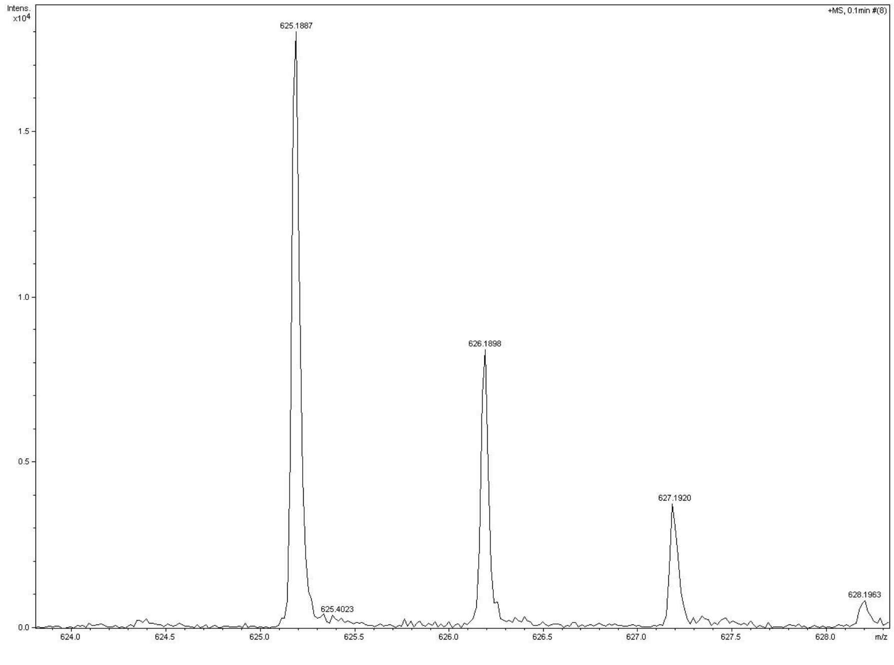

Dye-3

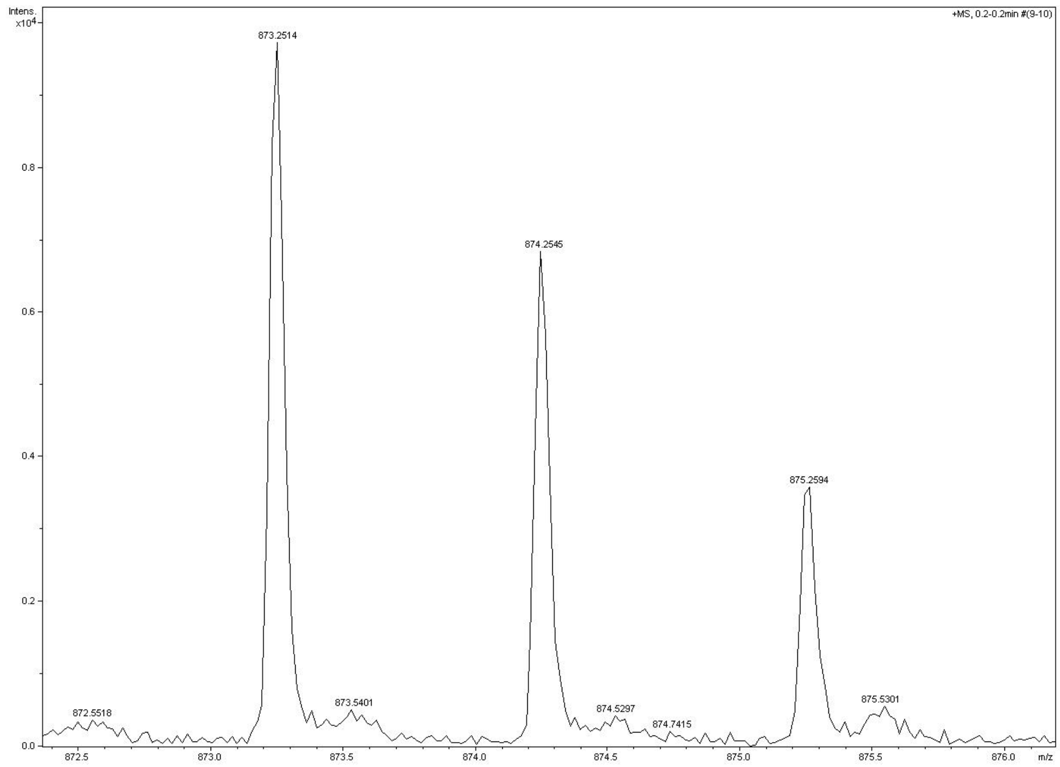
Intens.
x10^4
+MS, 0.2-0.2min #(9-10)
873.2514
874.2545
875.2594
872.5518
873.5401
874.5297
874.7415
875.5301
0.8
0.6
0.4
0.2
0.0
872.5
873.0
873.5
874.0
874.5
875.0
875.5
876.0
m/z

Dye-4

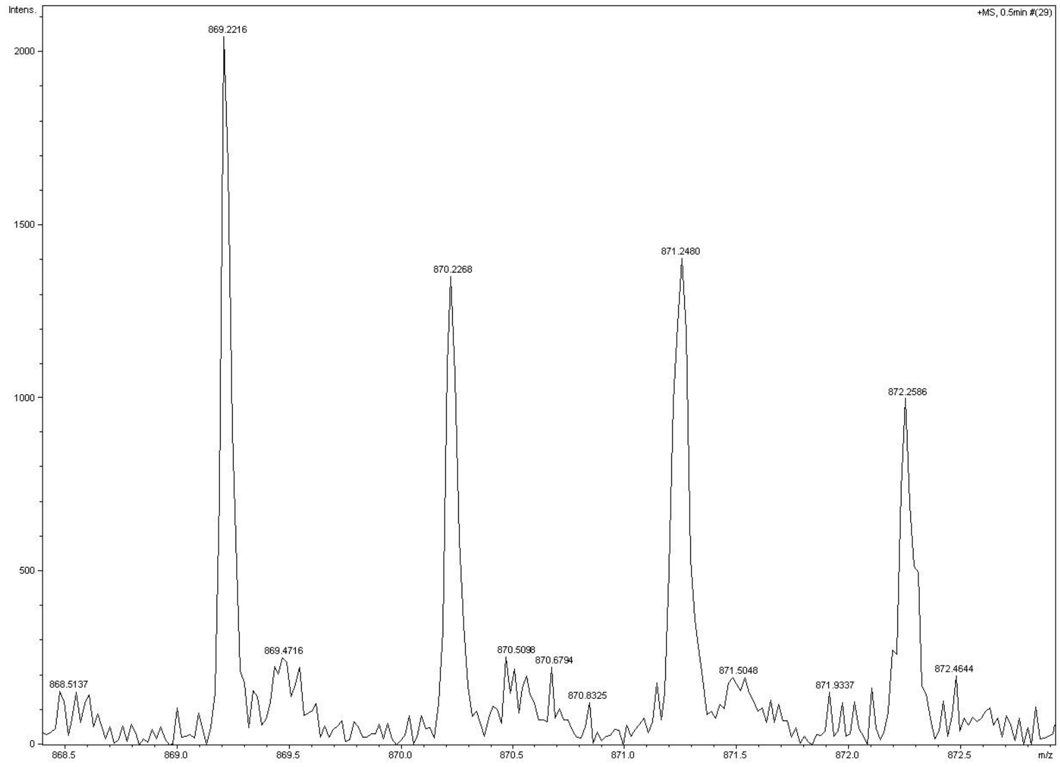
Intens.
+MS, 0.5min #(29)
869.2216
870.2268
871.2480
872.2586
868.5137
869.4716
870.5098
870.6794
870.8325
871.5048
871.9337
872.4644
2000
1500
1000
500
0
868.5
869.0
869.5
870.0
870.5
871.0
871.5
872.0
872.5
m/z

Dye-6

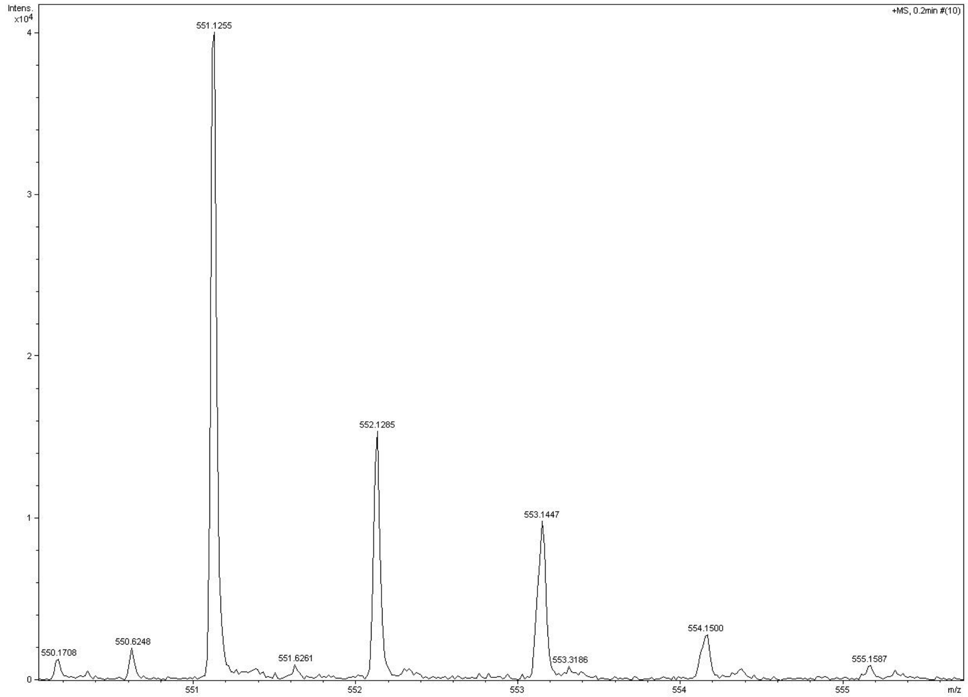
Intens.
x10^4
+MS, 0.2min #(10)
551.1255
552.1285
553.1447
550.1708
550.6248
551.6261
553.3186
554.1500
555.1587
4
3
2
1
0
551
552
553
554
555
m/z

Dye-7

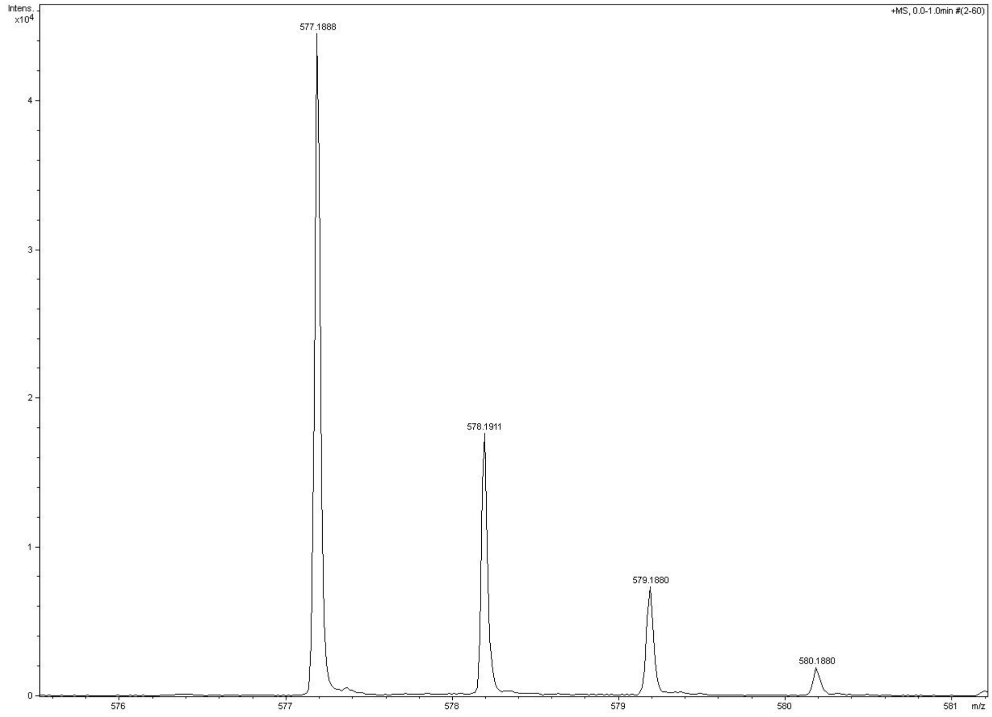
Intens.
x10^4
+MS, 0.0-1.0min #(2-60)
577.1888
578.1911
579.1880
580.1880
4
3
2
1
0
576
577
578
579
580
581
m/z

Dye-8

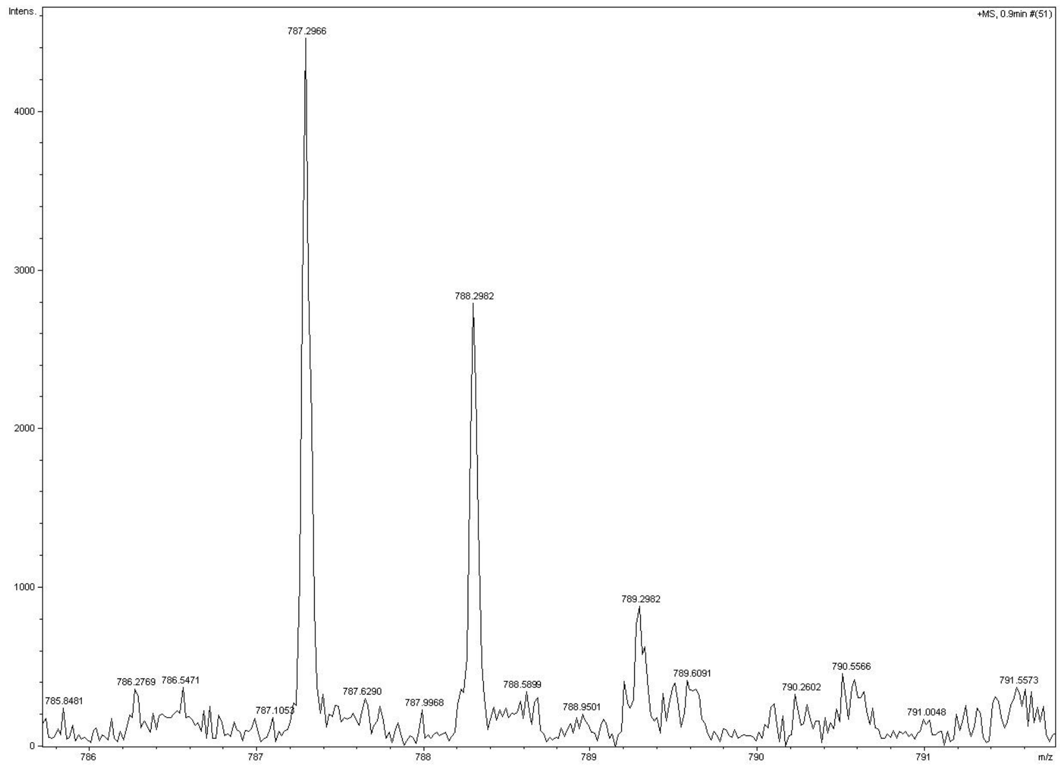

Intens.
+MS, 0.9min #(51)
787.2966
788.2982
789.2982
785.8481
786.2769
786.5471
787.1053
787.6290
787.9968
788.5899
788.9501
789.6091
790.2602
790.5566
791.0048
791.5573
4000
3000
2000
1000
0
786
787
788
789
790
791
m/z
